# Development and Validation of a Large Language Model–Powered Chatbot for Neurosurgery: Mixed Methods Study on Enhancing Perioperative Patient Education

**DOI:** 10.2196/74299

**Published:** 2025-07-15

**Authors:** Chung Man Ho, Shaowei Guan, Prudence Kwan-Lam Mok, Candice HW Lam, Wai Ying Ho, Calvin Hoi-Kwan Mak, Harry Qin, Arkers Kwan Ching Wong, Vivian Hui

**Affiliations:** 1 Neurosurgery Department Queen Elizabeth Hospital Kowloon China (Hong Kong); 2 Department of Electrical and Electronic Engineering Hong Kong Polytechnic University Kowloon China (Hong Kong); 3 Faculty of Medicine Chinese University of Hong Kong New Territories China (Hong Kong); 4 School of Clinical Medicine Li Ka Shing Faculty of Medicine University of Hong Kong Central and Western District China (Hong Kong); 5 Center for Smart Health School of Nursing Hong Kong Polytechnic University Kowloon China (Hong Kong); 6 School of Nursing Hong Kong Polytechnic University Kowloon China (Hong Kong); 7 Health and Community Systems School of Nursing University of Pittsburgh Pittsburgh, PA United States

**Keywords:** large language model, neurosurgery, chatbot, perioperative care, artificial intelligence, patient education, retrieval-augmented generation, digital health, patient-centered care

## Abstract

**Background:**

Perioperative education is crucial for optimizing outcomes in neuroendovascular procedures, where inadequate understanding can heighten patient anxiety and hinder care plan adherence. Current education models, reliant on traditional consultations and printed materials, often lack scalability and personalization. Artificial intelligence (AI)–powered chatbots have demonstrated efficacy in various health care contexts; however, their role in neuroendovascular perioperative support remains underexplored. Given the complexity of neuroendovascular procedures and the need for continuous, tailored patient education, AI chatbots have the potential to offer tailored perioperative guidance to improve patient education in this specialty.

**Objective:**

We aimed to develop, validate, and assess NeuroBot, an AI-driven system that uses large language models (LLMs) with retrieval-augmented generation to deliver timely, accurate, and evidence-based responses to patient inquiries in neurosurgery, ultimately improving the effectiveness of patient education.

**Methods:**

A mixed methods approach was used, consisting of 3 phases. In the first phase, internal validation, we compared the performance of Assistants API, ChatGPT, and Qwen by evaluating their responses to 306 bilingual neuroendovascular-related questions. The accuracy, relevance, and completeness of the responses were evaluated using a Likert scale; statistical analyses included ANOVA and paired *t* tests. In the second phase, external validation, 10 neurosurgical experts rated the responses generated by NeuroBot using the same evaluation metrics applied in the internal validation phase. The consistency of their ratings was measured using the intraclass correlation coefficient. Finally, in the third phase, a qualitative study was conducted through interviews with 18 health care providers, which helped identify key themes related to the NeuroBot’s usability and perceived benefits. Thematic analysis was performed using NVivo and interrater reliability was confirmed through Cohen κ.

**Results:**

The Assistants API outperformed both ChatGPT and Qwen, achieving a mean accuracy score of 5.28 out of 6 (95% CI 5.21-5.35), with a statistically significant result (*P*<.001). External expert ratings for NeuroBot demonstrated significant improvements, with scores of 5.70 out of 6 (95% CI 5.46-5.94) for accuracy, 5.58 out of 6 (95% CI 5.45-5.94) for relevance, and 2.70 out of 3 (95% CI 2.73-2.97) for completeness. Qualitative insights highlighted NeuroBot’s potential to reduce staff workload, enhance patient education, and deliver evidence-based responses.

**Conclusions:**

NeuroBot, leveraging LLMs with the retrieval-augmented generation technique, demonstrates the potential of LLM-based chatbots in perioperative neuroendovascular care, offering scalable and continuous support. By integrating domain-specific knowledge, NeuroBot simplifies communication between professionals and patients while ensuring patients have 24-7 access to reliable, evidence-based information. Further refinement and research will enhance NeuroBot’s ability to foster patient-centered communication, optimize clinical outcomes, and advance AI-driven innovations in health care delivery.

## Introduction

### Background

Neurosurgery is one of the specialties that have the highest demand in the medical field globally. An estimated 13.8 million patients require neurosurgical care annually worldwide [1]. The complex neuroanatomy and the wide variety of neurosurgical conditions often lead patients to have an array of surgical-related questions, fears, and concerns. Furthermore, up to 90% of neurosurgical patients report substantially preoperative anxiety, which has been associated with poorer perioperative outcomes. These patients often seek detailed information to make well-informed decisions about their health and treatment options. The need for comprehensive understanding affects not only their treatment choices but also the subsequent aftercare for both patients and their caregivers. Traditionally, face-to-face education supplemented by handouts such as brochures, pamphlets, and multimedia resources were the common ways to deliver health education. However, these approaches are labor-intensive, time-consuming, and inefficient within fast-paced clinical environment. Moreover, patients may not always receive the specific information they need or an immediate response to their concerns, leaving them feeling inadequately supported during critical decision-making.

With growing digital literacy, many patients now turn to online platforms for medical information, including support groups, consumer health websites (eg, MedlinePlus and WebMD), and social media platforms (eg, Facebook, Reddit, and Instagram) [2,3]. Hirvonen [4] delineated that the anonymity and disinhibiting effects of online help-seeking could allow greater patient autonomy, self-efficacy, and empowerment, enabling patients to make informed medical decisions. Nevertheless, these online health education materials, particularly in surgery specialties, often adopt a one-size-fits-all approach. Patients with complicated surgical needs and perioperative fears may find online health information to be irrelevant, unreliable, and not suitable for their specific needs and circumstances.

Perioperative care in neurosurgery is uniquely challenging due to the complexity of the procedures and the critical need for continuous, tailored patient education. Static resources—whether traditional or online—are often insufficient to meet the demand for tailored, real-time responses. This gap highlights the importance of innovative solutions that can deliver accurate, relevant, and timely information tailored to the unique needs of patients undergoing neurosurgery.

With the fast-growing technological development, the integration of artificial intelligence (AI) in the medical field is becoming increasingly prevalent, particularly in medical specialties. The interaction between AI and medicine has ushered in a new era of patient care and health information dissemination. Specialties such as anesthesiology, cardiology, and radiology have widely adopted AI to assist their daily consultations [5-7]. For example, in cardiology, the use of AI can help to analyze various cardiac imaging and predict coronary artery risk. In anesthesiology, the Philips IntelliVue monitor uses AI to continuously monitor vital signs and provide real-time alerts to anesthesiologists. In addition, AI technologies accurately analyze computed tomography and X-ray images to identify neurological pathologies, such as brain hemorrhages. While AI has been successfully implemented in other specialties, its application in neurosurgical perioperative care is particularly promising due to the high stakes and complexity of these procedures.

Beyond traditional imaging and monitoring, large language models (LLMs) have recently demonstrated remarkable performance in tasks requiring complex clinical reasoning, including answering standardized medical examinations. Recent work by Gajjar et al [8] evaluated the multimodal capabilities of GPT-4o on image-based United States Medical Licensing Examination Step 1, Step 2, and Step 3 examination questions, showing its potential to integrate visual and textual understanding in real-time clinical scenarios. These findings highlight the growing capacity of AI not only to support diagnostics but also to enhance medical education and patient communication.

In this regard, the increasing specialization and sophistication of AI applications in health care play a crucial role in meeting the patients’ diverse expectations. Platforms such as Neurosurgery Atlas and Copilot (ie, LLM-driven chatbots) are designated specifically for neurosurgery, demonstrating the growing demand for AI in facilitating the dissemination of medical information, addressing patient queries, and alleviating their concerns by providing timely and reliable responses based on patient characteristics. These chatbots can generate customized responses in an accessible format that addresses the unique concerns of each patient, which has demonstrated promising results in alleviating anxiety and improving overall patient engagement in the medical management process [9,10]. For example, Cevasco et al [11] reported that these advancements exemplify the paradigm shift toward patient-centered care that emphasizes transparency, shared decision-making, and patient autonomy.

Despite the promising features of LLM-driven chatbots, their implementation in clinical settings necessitates careful consideration of data privacy, information accuracy, and clinical relevancy. It has been demonstrated that the LLM-driven chatbots demonstrate high accuracy and completeness, but their readability scores may need improvement. Addressing these challenges will be crucial for ensuring the effectiveness and accessibility of LLM-driven chatbots for diverse patient populations in clinical settings.

However, little is known about the performance and clinical relevance of LLM-driven chatbots based on the domain experts’ evaluation. More importantly, there is a significant lack of clear understanding of its impact on clinical practice in neurosurgery. Without bridging this knowledge gap, it will be challenging to fully leverage LLM-driven solutions to enhance health care outcomes. The three research questions for this study are as follows: (1) How can the NeuroBot, using LLMs, be developed to optimize perisurgical education for neurosurgical patients? (2) What is the quantitative performance of the NeuroBot in terms of accuracy, relevance, and completeness? and (3) What are the perspectives of neurosurgical domain experts regarding the NeuroBot as a tool for patient education and support, and what is its potential for integration into clinical practice?

### Objectives

Through exploring these aspects, we aimed to examine the effectiveness of LLM-driven chatbots in neurosurgery and modify the NeuroBot based on domain experts’ feedback accordingly to foster advancement in patients’ care, medical training, and overall health care service quality.

## Methods

### Study Design

#### Overview

**Figure 1:**
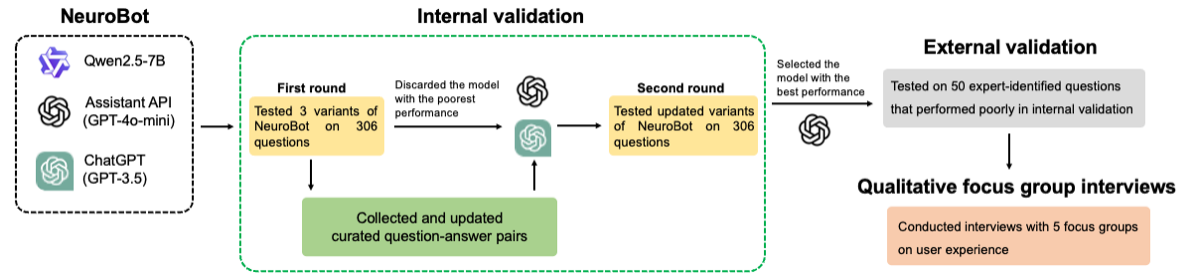
Mixed methods study design for NeuroBot evaluation.

This study used a convergent mixed methods design, integrating quantitative validation and qualitative focus group interviews to comprehensively evaluate the performance and clinical relevance of the NeuroBot (Figure 1). The quantitative component focused on internal and external validation of chatbot responses across domains using metrics of accuracy, relevance, and completeness. To complement this, the qualitative component included focus group interviews with neurosurgical domain experts, aimed at capturing user experiences, perceived utility, and practical considerations for clinical integration.

This design was selected to enable triangulation of findings, where objective chatbot performance could be validated and enriched by subjective expert perspectives. It also supported the identification of usability issues, contextual barriers, and refinement needs that may not be evident through quantitative assessment alone.

The integration of qualitative insights with quantitative performance data enabled a more nuanced understanding of the chatbot’s strengths and limitations, directly informing future design iterations and implementation strategies.

#### Internal Validation and External Validation

##### Overview

NeuroBot was developed using 3 LLMs: ChatGPT (GPT-3.5), Assistants API (GPT-4o-mini), and Qwen (Qwen2.5-7B), integrated with a retrieval-augmented generation (RAG) framework to ensure accurate, evidence-based neurosurgical information delivery. The knowledge base encompassed 8 domains: general hospital details, disease-related education, preoperative instructions, perioperative expectations, postoperative advice, wound care, medication guidance, and complication management. Although complete blinding was not feasible due to the exploratory nature of the pilot study, evaluators were instructed to independently assess chatbot responses using a standardized rubric, without knowledge of which model generated the output. To reduce bias, responses were anonymized, and participants provided both Likert-scale ratings and optional narrative justifications to allow for qualitative triangulation of scores.

##### Internal Validation

A total of 8 participants (n=6, 75% specialty-trained nurses and n=2, 25% final-year medical students) with expertise in neurosurgical patient education conducted the internal validation. This sample size was pragmatically chosen for structured model comparison rather than inferential analysis, and it enabled effective qualitative judgment across linguistic and domain contexts. Each model was assessed using the same curated set of 306 questions translated into both English and traditional Chinese. The questions, derived from real-world clinical encounters, spanned 8 perioperative domains (Table 1). Evaluators rated responses based on 3 predefined metrics: accuracy (1-6 scale), relevance (1-6 scale), and completeness (1-3 scale). The same set of questions and rating rubric were applied uniformly across all models. The model that achieved the highest average composite score across all 3 metrics—indicating superior performance in factual correctness, clinical relevance, and comprehensiveness—was selected for further refinement and external validation.

**Table 1:** Sample questions used for model training in each neurosurgery domain.

Domain	Number of questions	Sample questions
General neurosurgical questions	44	“What should I do if I need to reschedule my follow-up appointment?”
Disease-based questions	62	“What are the signs and symptoms of a ruptured aneurysm?”
Preoperative: preparation	34	“How long do I need to fast before surgery, and can I drink clear water?”
Perioperative	20	“What will I experience when I enter the operation theatre?”
Postoperative: general questions	48	“What symptoms should prompt me to contact my doctor after the procedure?”
Postoperative: wound management	26	“What are the wound care instructions for the puncture site?”
Postoperative: medication	40	“Are there any adverse effects that I need to be aware of when taking anticonvulsants?”
Postoperative: complication	32	“What should I do if I feel numbness or weakness in my limbs?”

A total of 918 responses were generated (306 questions×3 models), evaluated for consistency and bilingual performance. The traditional Chinese questions were collected from frontline nurses and physicians and were translated into English by a bilingual neurosurgical nurse. Back-translation was conducted by an independent bilingual physician to ensure semantic fidelity. Three bilingual neurosurgical professionals (including 2 nurse specialists and 1 nurse consultant) reviewed all question–response pairs for conceptual, linguistic, and cultural equivalence. Items not reaching consensus were revised through 2 structured discussions.

##### External Validation

The external validation phase followed the internal comparison of models and focused exclusively on the top-performing LLM configuration identified earlier (ChatGPT-based models). This phase aimed to evaluate the chatbot’s feasibility, practical relevance, and clinical accuracy through broader domain expert input. After selecting the best-performing LLM from the pilot study (internal validation), external validation was conducted with a broader group of domain experts, including 3 experienced nurses, 2 junior nurses, and 5 neurosurgeons. Participants were recruited from neurosurgical wards and high-dependency units. Recruitment criteria included direct involvement in neurosurgical patient care and the ability to provide expert feedback on the NeuroBot’s accuracy, usability, and relevance to clinical practice as shown in Textbox 1. The sample size was informed by qualitative research guidance, which indicates that expert usability assessments and validation studies typically reach thematic saturation with 5 to 30 participants [12]. In this study, a smaller sample size was intentionally adopted to enable a focused and in-depth exploration of participants’ lived experiences with NeuroBot, emphasizing its feasibility and practical use from the perspective of frontline health care providers.

**Participant recruitment criteria.**

- Experienced nurses (≥5 years of experience): their in-depth knowledge of neurosurgical care allowed them to critically assess NeuroBot’s functionality and practical applications.
- Junior nurses (<2 years of experience): their role in patient education and routine communication made them ideal for evaluating NeuroBot’s user-friendliness.
- Neurosurgeons (≥4 years of experience): their expertise in clinical decision-making and adherence to medical guidelines ensured a robust evaluation of NeuroBot’s accuracy and reliability.

Overall, 50 chatbot-generated responses were selected using purposive stratified sampling, drawing from the same 8 clinical domains, and were evaluated by a diverse panel including experienced neurosurgical nurses and neurosurgeons. These responses were selected to represent a distribution of performance levels (low: score 1-2, moderate: score 3-4, and high: score 5-6) as per prior internal ratings, ensuring a balanced assessment. The same 3 evaluation criteria—accuracy, relevance, and completeness—were used to maintain consistency in assessment methodology across both validation phases. The distribution of questions reviewed by experts is detailed in Table 2.

**Table 2:** Sample questions and evaluation performance in external validation.

Domain	Number of questions	Sample questions
General neurosurgical questions	4	“What kind of amenities should I prepare for the admission?” “Can I get all the investigation results/imaging report/medical for secondary opinion?”
Disease-based questions	8	“What is the advice or restrictions to my daily activities if I have an untreated and unruptured aneurysm?” “To treat cerebral aneurysms, besides endovascular interventions, what other treatment options are available?”
Preoperative preparation	6	“What are the essential pre-surgical preparations for embolization of cerebral aneurysm?” “The doctor said I need to undergo carotid stent placement surgery. I am a bit worried; is this surgery complicated and is it risky?”
Perioperative	6	“What is the procedure for endovascular embolization for cerebral aneurysms?” “Will I have any pain sensation during embolisation of AVM [arteriovenous malformation]?”
Postoperative: general questions	8	“What lifestyle changes or precautions will I need to take after the embolization procedure?” “When will I be allowed to mobilise in hospital e.g. get out of bed, after cerebral aneurysm embolisation surgery?”
Postoperative: wound management	6	“What if I found the puncture site dressing oozing with blood persistently after discharge to home?” “What are the wound care instructions for the puncture site?”
Postoperative: medication	6	“I have an unruptured AVM, and the doctor prescribed me with dilantin to control seizure. Should I increase the dosage myself if I have a more frequent seizure attack than usual?” “Do I need to continue current antiplatelets after carotid stent surgery?”
Postoperative: complication	6	“In what situation do I need to seek medical help after carotid stent surgery when discharged home?” “What is the most severe complication of aneurysm embolization surgery?”

#### Qualitative Study

We used inductive thematic analysis following 6-phase approach by Braun and Clarke [13]. Transcripts were analyzed iteratively, and initial codes were generated independently by 2 researchers. The coding framework was refined through consensus meetings, and a third reviewer validated the final themes. Thematic saturation was considered achieved when no new codes or insights emerged after the eighth transcript, indicating sufficient depth and coverage. Triangulation was used by comparing responses across professional groups (eg, experienced nurses and neurosurgeons) to enhance interpretive validity. Although formal participant validation (member checking) was not conducted due to time constraints, clinical stakeholders were consulted to ensure the thematic findings reflected practical realities in perioperative neurosurgical care.

Five focus group sessions were conducted, each lasting 30 to 40 minutes, within 1 week after participants completed an online performance evaluation questionnaire. The interviews were held in a neurosurgical office at Queen Elizabeth Hospital. Before starting the interview, the interviewer (CMH or WYH) introduced the study objectives briefly and collected the sociodemographic data of the participants. Interviews were facilitated using a semistructured interview guide composed of the following topics: (1) general perceptions of the NeuroBot, (2) usability and integration to routine care, (3) accuracy and relevance in clinical practice, (4) potential impact on patient care, and (5) challenges and recommendations. The questions for each topic are listed in Multimedia Appendix 1. The interviews were audio-recorded with participants’ consent and later anonymized and transcribed verbatim for analysis by the interviewer.

### Development Pipeline

#### Overview

The development of the NeuroBot followed a comprehensive pipeline, integrating advanced RAG technology with robust web development practices. This system allows patients to ask questions related to neurosurgery. In response, the NeuroBot provides accurate information sourced from our knowledge base, leveraging RAG technology. This section outlines the key components and processes involved in creating a reliable and effective NeuroBot for the patient.

#### Knowledge Base Development

The foundation of the NeuroBot’s knowledge base was established through a meticulous process of data collection and preprocessing. We sourced information from a variety of reputable neurosurgery resources, including online medical resources such as UpToDate, peer-reviewed journals, and publications from official health organizations. In addition, 6 nurses with 5 to 18 years of experience in the neurosurgery department contributed by generating pairs of frequently asked questions along with detailed answers. Given the use of Cantonese, we also provided comprehensive documentation outlining the accurate translation of medical terms between English and Cantonese to facilitate effective communication within the chatbot. This diverse range of sources, combined with careful attention to linguistic accuracy, ensured comprehensive coverage of neurosurgery topics relevant to patient inquiries.

Once collected, the data underwent a rigorous cleaning and curation process. This involved removing outdated or irrelevant information, standardizing medical terminology to ensure consistency, and verifying the accuracy of the content. We paid particular attention to aligning our information with the latest medical guidelines and best practices.

To enhance the utility of our data for the RAG system, particularly with academic papers, textbooks, and other free-text sources, we used an approach that used GPT-3.5 to generate question-answer pairs based on the original documents. This process involved extracting key information from complete PDF documents and other textual resources. We then used GPT-3.5 to formulate relevant questions that patients might ask, along with comprehensive answers derived from those sources.

In addition to generating question-answer pairs from the documentation, we also used GPT-3.5 for data augmentation. We requested it to create variations of the same questions to ensure that the chatbot could effectively understand inquiries, regardless of how users phrased them. After generating these question-answer pairs, our nurses reviewed them to verify the accuracy and appropriateness of the responses. We removed any incorrect pairs or made necessary corrections.

**Figure 2:**
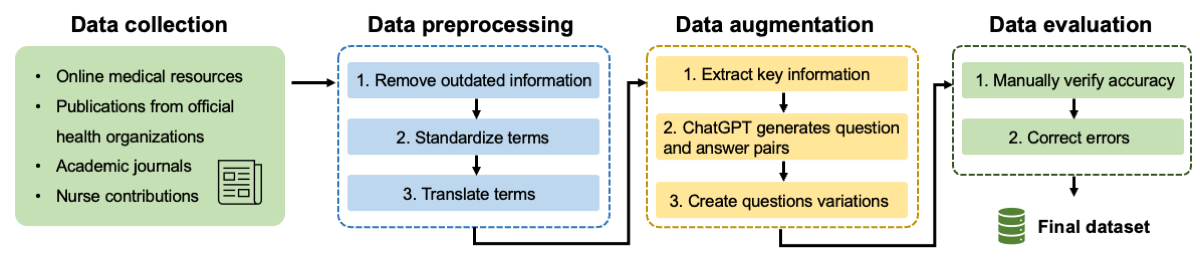
The data handling process of NeuroBot.

Finally, the validated question-answer pairs were converted into a JSON format, which facilitates easier processing by our retrieval system. The entire data handling process is illustrated in Figure 2. This data preparation step was essential in bridging the gap between raw neurosurgery information and the types of queries patients typically pose. By anticipating potential questions and providing preformulated answers, we developed a wide and comprehensive knowledge base in neurosurgery. This significantly enhanced the NeuroBot’s capability to deliver relevant and accurate responses to a diverse array of neurosurgery inquiries. The entire knowledge base has set to be updated according to the neurosurgery specialty usual practice, usually between 6 months to 1 year.

#### RAG Process

The RAG process forms the core of the NeuroBot’s ability to provide accurate and contextually relevant responses. RAG combines the power of LLMs with the precision of information retrieval systems, resulting in a chatbot that can generate humanlike responses while grounding them in factual medical information [14].

At its core, RAG operates on a 2-step principle: retrieval and generation [15]. When a user submits a query, the system first retrieves relevant information from our curated knowledge database. This retrieval step uses advanced semantic similarity calculation to identify the most pertinent documents or passages related to the user’s question [16]. Once relevant information is retrieved, it is passed to the generation component along with the original query. This is where the power of the language model comes into play. The model, whether it is the GPT-3.5 or the Qwen model, takes the retrieved information as context and generates a response that addresses the user’s query. This generation process ensures that the response is not merely a regurgitation of stored information but a coherent, natural-language answer that addresses the user’s needs.

In this research, we used 3 different methods, including direct use of ChatGPT (GPT-3.5 based), the File Search tool of the OpenAI Assistants API (GPT-4o-mini based), and Qwen, to implement the RAG process. For ChatGPT, which we accessed directly through the OpenAI interface, we uploaded our knowledge base to the GPT-3.5 model. The system automatically processed the uploaded files and performed RAG when it received queries. Similarly, with the File Search tool of OpenAI Assistants API, we followed the same principle; however, the interaction occurs through the API. We uploaded the files and submitted queries programmatically, allowing for more flexible integration into our systems. According to OpenAI documentation [17], the Assistants API supports various features that enhance the RAG process, including rewriting user queries to optimize them for search, breaking down complex queries into multiple parallel searches, conducting both keyword and semantic searches across assistant and thread vector stores, and reranking search results to select the most relevant responses before generating the final output. For the Qwen model, we implemented RAG by using its built-in retrieval capabilities. This involved integrating a custom retrieval system that queried our knowledge base and retrieved relevant documents before generating responses. By leveraging Qwen’s architecture, we ensured that the model could efficiently access and use the information stored in our knowledge base, thereby providing accurate and contextually relevant answers to patient inquiries.

#### User Interface

**Figure 3:**
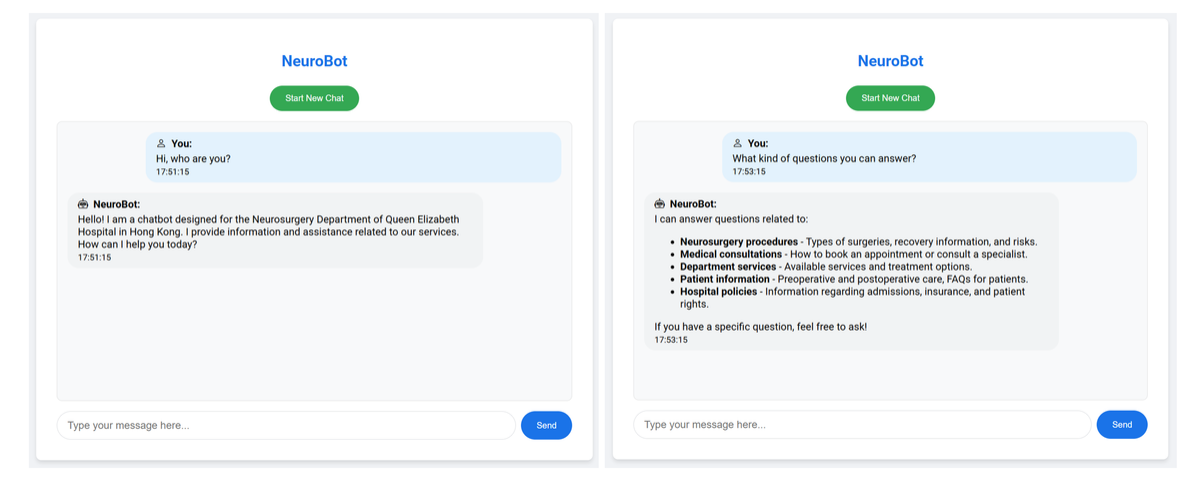
NeuroBot interface: patient information query use cases.

As shown in Figure 3, the user interface of the NeuroBot was developed using Django, a robust web framework that supports scalable and responsive application design. The interface is structured to be intuitive, readable, and mobile-responsive, with a clean layout and labeled input fields that guide users in entering their neurosurgical questions. Key design features include (1) responsive design; it is compatible with both desktop and mobile formats to ensure accessibility, (2) real-time interaction; it supports instant response generation and feedback cues (eg, loading spinners and confirmation messages), and (3) clear visual cues; feedback elements such as colored status indicators and readable text formatting aid usability.

While formal usability testing using validated heuristics (eg, Nielsen’s usability principles) was not performed during this initial phase, the interface underwent iterative refinement based on feedback from neurosurgical nurses and physicians. These health care professionals evaluated the interface during pilot deployment and provided comments on usability, clarity, and navigational logic. Their feedback informed modifications to font size, button labeling, and content alignment, improving user engagement.

Given the importance of layout readability and cognitive accessibility in digital health tools—particularly for patients with cerebrovascular disease—we prioritized a low cognitive load design. As highlighted in recent literature [8], optimizing interface design is critical to support patient understanding and adherence. Future iterations of NeuroBot will incorporate formal usability testing with target patient groups to further refine the platform and ensure alignment with best practices in digital health communication. By leveraging Django’s features and capabilities, we created a robust and efficient interface that enhances user engagement and satisfaction with the NeuroBot.

### Participant Recruitment and Data Collection

Participants include neurosurgeons and nurses with expertise in neurosurgery. Neurosurgeons are actively practicing and proficient in English and Chinese, with at least 3 years of clinical experience and completed fellowship training. For nurses, they must be registered in the Hong Kong Nursing Council, have at least 2 years of neurosurgical experience, be proficient in English and Chinese, and possess advanced neurosurgical specialty training. The qualitative study recruited participants from the quantitative arm of this study and additionally included several higher specialist trainees and neurosurgical nurses with at least 1 year of experience in neurosurgery to gather diverse insights from frontline professionals.

Participants were recruited on a voluntary basis as invited by interviewers whether they were interested in “taking part in a project that will test an AI chatbot.” All participants were recruited from the neurosurgical department at Queen Elizabeth Hospital in Hong Kong. They were given 1 week before the interview to interact with NeuroBot by inquiring about neurovascular disease and surgery-related questions.

Accuracy is evaluated on a 1 to 6 Likert scale to measure the factual correctness of NeuroBot’s responses, ensuring that the information provided is precise and trustworthy. Relevance is similarly assessed using a 1 to 6 Likert scale, focusing on the appropriateness of the chatbot’s responses in addressing the patient’s perioperative care needs. Completeness, however, is evaluated using a 1 to 3 Likert scale to reflect the categorical nature of this dimension, that is, whether responses are complete, partially complete, or incomplete. This scale was selected based on pilot testing with domain experts, who reported greater clarity and interrater agreement when assessing procedural and instructional content using this simplified framework. A broader 6-point scale introduced ambiguity in interpreting gradation levels and reduced scoring consistency. Moreover, the 3-point scale more directly captures the practical impact on patient comprehension, as even partially complete information may result in misunderstandings or misinformed decisions during perioperative care. These metrics—accuracy, relevance, and completeness—are the primary measures for evaluating NeuroBot’s performance in providing reliable and patient-centered perioperative support. While automated retrieval metrics such as top-k recall, precision, and retrieval accuracy can provide valuable insight into system performance, we prioritized a clinically meaningful evaluation approach in this study. Specifically, each generated response—based on its retrieved content—was manually reviewed and scored by domain experts for relevance, accuracy, and completeness. This allowed us to directly assess the practical effectiveness of the RAG process in producing appropriate and clinically useful answers. We believe this method offers a more robust and contextually relevant evaluation in health care settings than relying solely on retrieval metrics, which may not fully capture the quality or safety of the information delivered to patients.

### Statistical Analysis

#### Quantitative Statistical Analysis

The score results were presented descriptively, including mean (SD) values and 95% CIs. Comparisons among the 3 chatbot models (ChatGPT, Assistants API, and Qwen) were conducted using one-way ANOVA. For pairwise comparisons between 2 chatbot models (ChatGPT and Assistants API), a paired *t* test was applied. A 2-sided *P*<.05 was considered statistically significant. In the external validation by domain experts, a single chatbot model was evaluated using descriptive statistics, mean (SD) values and a 95% CI. The interrater agreement was evaluated using the intraclass correlation coefficient.

#### Qualitative Data Analysis

The interview transcripts were analyzed using an inductive thematic approach to identify common themes and subthemes related to the participants’ experiences with the NeuroBot [18]. Two independent reviewers, CMH and WYH, identified and developed key themes as patterns emerged from the data [19]. For the coding process and development of the codebook, NVivo (Lumivero) was used to systematically organize and categorize the data and tracking of recurring patterns within the qualitative data. The initial coding was followed by a discussion between the reviewers to resolve any discrepancies. To measure the consistency of coding between the 2 reviewers, Cohen κ was calculated to assess interrater reliability. A κ value of 0.78 was achieved, indicating a substantial level of agreement between the reviewers. Disagreements were resolved through consensus to ensure accuracy and completeness in the final coding structure.

### Ethical Considerations

The research was granted ethics approval in the Hospital Authority after review by the Central Institutional Review Board (CIRB-2024-486-3) and is also applying ethics approval in the Hong Kong Polytechnic University.

All participants involved in the internal and external validation, as well as the qualitative interviews, provided informed consent before participation. Participants were informed of the study’s purpose, their right to withdraw at any time without consequences, and how their data would be used and stored.

To protect participant privacy, all personal identifiers were removed from the dataset. Responses were anonymized and stored securely in password-protected files accessible only to the research team. No identifiable information was collected or retained during chatbot interaction evaluations or focus group interviews.

Participants were not provided with financial or material compensation for their involvement in the study. Their participation was entirely voluntary.

This research complies with the Declaration of Helsinki and conforms to the data protection guidelines outlined by the local governing bodies.

## Results

### Internal Validation

#### Overview

Our research incorporated 2 rounds of internal validation to evaluate the performance of the LLMs. In the first round, we assessed 3 models: ChatGPT, Assistants API, and Qwen. Following this initial validation, we discarded the model with the poorest performance. During the initial internal validation, researchers will evaluate the responses generated by the NeuroBot and document any clearly incorrect or ambiguous answers. These identified questions and responses will then be reviewed by domain experts, who will provide the correct answers. To address the identified shortcomings, we updated the NeuroBot’s database by adding relevant documents and curated question-answer pairs. After these updates, we performed a second round of internal validation on the remaining models—ChatGPT and Assistants API—to assess the impact of the changes. Responses were rated for accuracy and relevance on a 6-point scale (1=completely incorrect, 6=completely accurate and relevant), and for completeness on a 3-point scale (1=incomplete, 3=fully complete).

#### First Evaluation

In the initial evaluation, the Assistants API achieved a mean accuracy score of 5.28 (SD 1.48; 95% CI 5.11-5.45), a mean relevance score of 5.46 (SD 1.19; 95% CI 5.31-5.61), and a mean completeness score of 2.61 (SD 0.67; 95% CI 2.52-2.70) across the 306 chatbot-generated answers as shown in Table 3. ChatGPT recorded a mean accuracy score of 4.98 (SD 1.44; 95% CI 4.80-5.16), a mean relevance score of 5.28 (SD 1.04; 95% CI 5.15-5.41), and a mean completeness score of 2.79 (SD 0.56; 95% CI 2.72-2.86). In contrast, Qwen underperformed with a mean accuracy score of 2.63 (SD 1.38; 95% CI 2.51-2.85), a mean relevance score of 2.58 (SD 1.28; 95% CI 2.42-2.74), and a mean completeness score of 1.35 (SD 0.66; 95% CI 1.27-1). Due to its relatively poor performance, Qwen was excluded from further evaluation.

**Table 3:** Comparative evaluation of NeuroBot: Assistants API versus ChatGPT versus Qwen in the first round of validation.

Outcomes measured and model			Scores, mean (SD; 95% CI)		*P* value	
**Accuracy (1-6 scale)**						.29
	Assistants API	5.28 (1.48; 5.11-5.45)				
	ChatGPT	4.98 (1.44; 4.80-5.16)				
	Qwen	2.63 (1.38; 2.51-2.85)				
**Relevancy (1-6 scale)**						.63
	Assistants API	5.46 (1.19; 5.31-5.61)				
	ChatGPT	5.28 (1.04; 5.15-5.41)				
	Qwen	2.58 (1.28; 2.42-2.74)				
**Completeness (1-3 scale)**						.06
	Assistants API	2.61 (0.70; 2.52-2.70)				
	ChatGPT	2.79 (0.56; 2.72-2.86)				
	Qwen	1.35 (0.66; 1.27-1.43)				

#### Second Evaluation

After addressing translation issues and occasional incomplete wordings identified in the first round by incorporating the incorrect questions and answers into the database, a second evaluation was conducted on ChatGPT and the Assistants API. In the second round, the Assistants API demonstrated consistent results with a mean accuracy score of 5.28 (SD 1.02; 95% CI 5.21-5.35), a mean relevance score of 5.17 (SD 0.92; 95% CI 5.10-5.24), and a mean completeness score of 2.43 (SD 0.53; 95% CI 2.39-2.47) as shown in Table 4. ChatGPT slightly improved with a mean accuracy score of 4.98 (SD 1.10; 95% CI 4.90-5.06), a mean relevance score of 4.88 (SD 1.16; 95% CI 4.80-4.96), and a mean completeness score of 2.62 (SD 0.51; 95% CI 2.58-2.66).

**Table 4:** Comparative evaluation of NeuroBot Assistants API and ChatGPT performance in the second round of validation using standardized neurological assessment metrics.

Outcomes measured and model		Scores, mean (SD; 95% CI)	*P* value	
**Accuracy (1-6 scale)**				<.001
	Assistants API	5.28 (1.02; 5.21-5.35)		
	ChatGPT	4.98 (1.10; 4.90-5.06)		
**Relevancy (1-6 scale)**				<.001
	Assistants API	5.17 (0.92; 5.10-5.24)		
	ChatGPT	4.88 (1.16; 4.80-4.96)		
**Completeness (1-3 scale)**				<.001
	Assistants API	2.43 (0.53; 2.39-2.47)		
	ChatGPT	2.62 (0.51; 2.58-2.66)		

#### Subgroup Analysis of Assistants API and ChatGPT

For the Assistants API, of the 918 responses, 501 (54.6%) responses achieved the highest accuracy score of 6.0, while 264 (28.8%) responses were rated as nearly correct (accuracy score of 5.0). Only 5 (0.5%) responses were completely incorrect (accuracy score of 1.0). Most inaccurate responses, scoring ≤3 (n=64, 7.1%), pertained to preoperative and wound-related questions. In terms of relevance, 46.3% (425/918) of the responses received the highest relevance score of 6.0, and 30.5% (280/918) of the responses were nearly all relevant (relevance score of 5.0). Furthermore, 0.4% (4/918) of the responses were entirely irrelevant (relevance score of 1.0). For completeness, 44.7% (410/918) of the responses were rated as comprehensive, 53.7% (493/918) of the responses were rated as adequate, and 1.6% (15/918) of the responses were rated as incomplete.

For ChatGPT, among its 918 responses, 371 (40.4%) attained the highest accuracy score of 6.0, while 297 (32.3%) were rated as nearly correct (accuracy score of 5.0). Only one response (0.11%) was entirely incorrect (accuracy score of 1.0). Most inaccurate responses, scoring 3.0 or lower (95/918, 10.3%), were related to general hospital information and postoperative complications. In addition, 35.6% (326/918) of the responses were rated at the highest level of relevance (relevance score of 6.0), with 35.9% (330/918) of the responses rated as nearly all correct (relevance score of 5.0). Seven responses (0.8%) were completely incorrect (relevance score of 1.0). Of the 918 responses, 579 (63.1%) were classified as comprehensive, 326 (35.6%) as adequate, and 12 (1.3%) as incomplete.

Overall, the Assistants API outperformed ChatGPT in terms of accuracy and relevance. In addition, given its flexibility for integration across various social platforms, the Assistants API was selected as the preferred model for NeuroBot and has been updated for further external validation.

### External Validation

The mean (SD) accuracy score for the rescored answers was 5.70 (0.68), with 95% CI 5.46-5.94. The mean (SD) relevance score was 5.58 (0.6), with 95% CI 5.45-5.94. The mean (SD) completeness score was 2.70 (0.12), with 95% CI 2.73-2.97 as shown in Table 5. Among the original answers that had received an accuracy score of <3.0 (n=26), 24 (93.3%) showed improved accuracy scores upon rescoring, 2 (7.7%) questions remained the same, which is a question regarding preoperative preparation. For the original answers that received an accuracy score of 6.0 (n=24), 22 (93.3%) retained their perfect score, while 2 (8.3%) were downgraded to an accuracy score of 3.0, which is a question related to successful rate comparing embolization versus clipping surgery. There was strong interrater agreement, with intraclass correlation coefficient values of 0.981 for accuracy (*P*<.001), 0.955 for relevance (*P*<.001), and 0.970 for completeness (*P*<.001), which indicates excellent agreement among raters.

**Table 5:** External validation results for the latest version of NeuroBot Assistants API on independent neurological datasets.

Outcomes measured	Scores, mean (SD; 95% CI)
Accuracy (1-6 scale)	5.70 (0.68; 5.46-5.94)
Relevance (1-6 scale)	5.58 (0.6; 5.45-5.94)
Completeness (1-3 scale)	2.70 (0.12; 2.73-2.97)

### Thematic Analysis

After the external validation, 8 more health care staff were recruited to participate in the interviews. There were a total of 18 participants, including 11 (61%) nurses and 7 (39%) physicians, who took part in the focus group interviews as outlined in Table 6. They averagely spent approximately 0.5 to 1 hours with NeuroBot before the interview.

**Table 6:** Description of the participants (N=18).

Variables and subgroups		Participants, n (%)
**Age range (y)**		
	20-29	4 (22)
	30-39	11 (61)
	40-49	3 (17)
	≥50	0 (0)
**Sex**		
	Male	9 (50)
	Female	9 (50)
**Profession**		
	Physician	7 (39)
	Nurse	11 (61)
**Years of neurosurgery practice**		
	0-3	3 (17)
	4-9	5 (28)
	≥10	10 (56)
**With neurosurgical specialty nursing qualification or attained fellowship in neurosurgery**		
	Yes	13 (72)
	No	5 (28)
**Previous experience with chatbot**		
	Yes	11 (61)
	No	7 (39)

Five main themes were identified, which were further categorized into subthemes as shown in Table 7. The detailed analysis of each quote from domain experts is provided in Multimedia Appendix 2.

**Table 7:** Themes identified to explore the perception from domain experts on NeuroBot.

Main themes and subthemes		Sample quotes from domain experts
**Chatbot use as a patient resource tool**		
	Provide quick, reliable, and accessible information	“It’s helping them by providing prompt, validated and reliable information with 24/7 availability.”
	Save time and effort in finding needed details	“[C]hatbot can help by delivering accurate information almost instantly, saving patients the time and effort of searching on their own”
	Deliver tailored content for individual health literacy	“[I]t instantly regenerated responses using more simplified words and with more explanation.”
**Chatbot use as a health care tool enhancing health-related outcomes of patients**		
	Enhance patients’ psychological health	“NeuroBot can give them the information they need, which helps calm their nerves and makes the whole process a lot clearer for them.”
	Improve treatment compliance and reduce complications	“digital tools...to reinforce important information as reminders from time-to-time.”
	Boost patient self-efficacy	“[O]ffering preoperative tips and advice for when they’re back home, empowering them to manage their health.”
**Chatbot use as a patient-health care professional communication tool**		
	Offer a safe, anonymous space for personal concerns	“A patient wanted to know more about hospice and palliative care arrangements, but he said he did not dare to ask such issues in front of parents and his wife.”
	Aid junior staff in explaining complex neurosurgical concepts	“Explaining terms like arteriovenous malformation to patients in Cantonese is tough, but NeuroBot helps me clarify them quickly and accurately.”
	Save time on addressing general queries	“Explaining terms like arteriovenous malformation to patients in Cantonese is tough, but NeuroBot helps me clarify them quickly and accurately.”
	Bridge communication gaps between patients and staff	“My patients often forget their questions until after the doctor leaves. I think the bot could help bridge this communication gap.”
**Perceived preferred features of NeuroBot**		
	Simple, user-friendly design with natural language understanding	“It’s really easy to use, which is great for people who aren’t familiar with chatbot.”
	Provide quick, accurate, and patient-centered information	“The answers are clear, easy to understand, and cover common patient questions.”
**Perceived cons or concerns of NeuroBot**		
	Nonattractive design	“The design looks a bit too plain.”
	Concise content needed	“The info comes in long paragraphs, which can be hard to read.”
	Lack of voice or image inputs	“Adding features like voice input and output with voice and video, in addition to text would be better.”
	Public acceptability	“Patients and families might have concerns...might find it difficult to accept talking to a robot.”
	Information update	“It’s a big challenge to make sure the bot provides up-to-date information consistently.”
	Liability and ethical concerns	“If the information from the bot is wrong, it could be detrimental and who will bear the liability?”

## Discussion

### Principal Findings

Overall, our study illustrated the high level of feasibility of using LLMs to develop a chatbot for patient education. Our best model using the Assistants API revealed a high mean of 5.28/6 for accuracy and a high mean of 5.17/6 for relevancy. Our second-best model using ChatGPT still obtained a high mean of 4.98/6 for accuracy and a high mean of 4.88/6 for relevancy. Independent external validation was done by multiple domain experts with years of experience in the field yielding a strong interrater agreement. The external validation also yielded a high mean accuracy score of 5.70/6, a high relevance score of 5.58/6, and a high completeness score of 2.70/3, as well as a statistically significant improvement from our internal scoring in all 3 areas, revealing that the internal scorings by our team members were highly stringent regarding all 3 areas. This demonstrates that with extensive training and fine-tuning, it is possible to develop an LLM that provides satisfactory responses to inquiries regarding neurosurgical endovascular conditions.

Table 7 summarizes the 5 primary qualitative themes derived from the interviews, which include (1) chatbot use as a patient resource tool, (2) chatbot use as a health care tool enhancing health-related outcomes of patients, (3) chatbot use as a patient-health care professional communication tool, (4) perceived preferred features of NeuroBot, and (5) perceived cons or concerns of NeuroBot. The identified themes from our qualitative evaluation reflect stakeholder perspectives on the functional and relational roles of chatbots in perioperative care.

Another strength of the NeuroBot lies in its use of a RAG framework, which enhances the accuracy and contextual relevance of its responses. By combining LLMs with a domain-specific retrieval system, the NeuroBot is able to produce humanlike responses that are grounded in verified medical knowledge. A critical concern when deploying LLMs in health care contexts is the potential for hallucinations—instances where the model generates responses that are plausible-sounding but factually incorrect or fabricated [20]. To address this, we implemented several mitigation strategies. First, the reliance on retrieval from a curated, domain-specific knowledge base ensures that responses are consistently anchored in trusted sources. Second, during the internal validation process, domain experts systematically reviewed model outputs to identify and correct hallucinated or ambiguous responses. These insights were used to iteratively refine the knowledge base, enhancing the alignment between retrieved content and generated responses. In addition, question-answer pairs derived from problematic cases were incorporated into the system to improve performance on similar queries in the future. These measures collectively contributed to reducing hallucinations and improving the reliability of the chatbot’s outputs.

### Comparison With Literature

Other research has been previously done to investigate the use of LLMs in patient communication, including fields such as obstetrics [21], nephrology [22], general medicine [23], and ophthalmology [24]. Gilson et al [24] created an LLM with an RAG pipeline similar to ours on ophthalmology domain-specific knowledge and subsequently evaluated the LLM’s performance on the factuality of evidence, selection and ranking of evidence, attribution of evidence, and answer accuracy and completeness. On a 5-point scale, LLM with RAG obtained an average accuracy score of 3.23 and an average completeness score of 3.27, with a consequential amount of hallucination.

While it may seem on the surface that our research has obtained a better accuracy and completeness score than the previous LLM on ophthalmology [24], it should be careful to compare these metrics as they are subjective scores instead of objective metrics. Further studies will have to standardize the evaluation metrics for easy comparison of LLMs’ performance between 2 different studies. Myllyaho et al [25] highlighted that the benefit of inviting expert opinions to evaluate the AI model is that it provides insight into the usability and severity of potential issues from the perspective of those who encounter them in real-world situations. In addition, Markus et al [26] highlighted the need for external validation to ensure trustworthiness in AI models, particularly in health care applications. Involving health care providers in validating the chatbot helped increase transparency and build trust in the potential use of AI chatbots in clinical settings in the future.

Therefore, we attempted to verify our subjective scoring with external validation, which yielded a high interrater agreement, validating our 3 metrics, which have not been done in the aforementioned ophthalmology research [24]. Yet, our domain experts pointed out the importance of evidence attribution in medical applications and evaluated their LLM on this aspect. Further investigation can be done to evaluate our LLM on evidence of attribution in subsequent research.

Recent studies have further demonstrated the advanced capabilities of multimodal LLMs such as GPT-4o, which can process both text and medical images [27-29]. Notably, Gajjar et al [8] evaluated ChatGPT-4o’s performance on image-based questions from the United States Medical Licensing Examination Step 1, Step 2, and Step 3 examinations, showing that the model can achieve high levels of accuracy across complex clinical reasoning tasks. This frontier work reinforces the growing role of LLMs in clinical education and diagnostic reasoning, and supports the potential of conversational agents such as NeuroBot in managing nuanced patient interactions in neurosurgical care.

Unlike the study by Gajjar et al [8], our research focused on a clinically integrated chatbot specifically designed for perioperative neurosurgical education, leveraging a RAG framework. NeuroBot was fine-tuned using domain-specific knowledge and validated through structured internal and external expert reviews. Furthermore, NeuroBot offers bilingual support (English and traditional Chinese), enhancing accessibility for diverse patient populations in our local health care setting. In contrast to the generic ChatGPT models evaluated in the study by Gajjar et al [8], NeuroBot is embedded with procedural timelines, personalized responses, and interactive prompts designed to support the full perioperative journey. These features position NeuroBot as a task-specific, clinically validated tool tailored to neurosurgical patient needs, rather than a general-purpose AI responder.

### Clinical Implications

For medical professionals, integrating a domain-specific LLM such as NeuroBot streamlines patient communication amidst growing clinical workload. Most participants in our study—comprising neurosurgical clinicians and digital health experts—recognized NeuroBot’s potential as a valuable health care tool to enhance health-related outcomes. By addressing frequently asked questions (eg, preoperative instructions, ward logistics, and discharge care), the chatbot can alleviate frontline staff burden, allowing clinicians to concentrate on more complex and urgent tasks.

For patients, NeuroBot serves as an easily accessible and timely resource tool, delivering accurate and tailored perioperative information. Participants emphasized its ability to mitigate reliance on unvetted online sources and improve comprehension of neurosurgical procedures. This aligns with emerging evidence supporting LLM-based chatbots in enhancing health literacy and self-management in surgical patients.

From our qualitative evaluation, we found that health care providers viewed NeuroBot not only as an educational adjunct but also as a communication bridge between patients and health care providers. While not a substitute for clinical interaction, the chatbot was seen as a valuable supplement to improve patient–clinician dialogue and reinforce key information across the care continuum.

However, concerns were raised regarding the potential hesitancy among patients to trust or use chatbot technologies. To address this, we propose several strategies to build patient confidence and encourage adoption. Patient education campaigns can demystify AI applications and illustrate their benefits in health care. Clinician endorsement—whether through verbal recommendation or integration into patient consultations—can improve trust and legitimacy. Prior research suggests that trust in digital tools significantly increases when introduced by trusted health care professionals [30,31]. In addition, seamless integration of NeuroBot into clinical workflows and hospital systems may help normalize its use and enhance long-term engagement.

Finally, participants expressed interest in expanded functionality, such as greater personalization, multimedia support, and emotional responsiveness. The development of multimodal LLMs capable of incorporating visual aids or interactive elements may further enhance patient engagement and learning, particularly in the context of complex neurosurgical procedures. These directions hold promise for future iterations of NeuroBot.

### Limitations and Future Directions

In terms of limitations of the study, several sources of potential bias must be acknowledged. First, members of the development team participated in the internal validation phase, which may introduce confirmation bias. However, the external validation was conducted independently by experienced domain experts with no involvement in model development. Second, the absence of formal blinding may have allowed implicit bias to influence ratings; standardized scoring rubrics and independent evaluations were used to mitigate this. Third, while Likert-scale ratings provide structured assessment, they may be subject to social desirability bias. To address this, participants were anonymized and encouraged to offer critical feedback and explanatory comments alongside their scores.

Regarding the constraint of the current version of NeuroBot, it is the scope of the domain-specific knowledge. In this study, we chose to limit NeuroBot’s domain scope to neurosurgical endovascular diseases and surgeries. Our target for future study is to expand at least its domain to include all neurosurgical diseases that we commonly encounter, as well as all neurosurgical surgeries that are viable in our neurosurgical center to provide all-encompassing assistance to patients in our hospital. This goal will require our LLM to expand from the narrow domain of endovascular neurosurgery to a much wider domain of neurosurgery. One concern is the possibility of underfitting, where the current size of parameters of our LLM model may not be able to achieve satisfactory performance without upsizing the model. We plan to train our current model further on data from different fields of neurosurgery and assessing the performance of the model.

Future research could investigate the generalizability of the NeuroBot in adapting to other neurosurgical centers or even other specialties. The scope of use of our current NeuroBot is limited to our hospital due to the ground truth we provided. Information such as protocols, ward location, or facilities are specific to our current clinical site and will not be applicable to other clinical sites. However, we hypothesize that by using the RAG approach, our model can easily be adapted to other centers by swapping out the documents in the RAG vector embedding space and retraining the model. In addition, NeuroBot is specifically designed for the field of neurosurgery, incorporating a domain-specific database relevant to this area. However, the underlying development methodology remains consistent, allowing for easy adaptation to other medical specialties by modifying the content of the knowledge base. Given our structured data handling procedures and the use of the RAG framework, the system has the potential to perform well in other domains as well. Future researchers are encouraged to apply our approach to develop chatbots for different medical fields and conduct corresponding evaluations.

In addition, NeuroBot is currently evaluated by trained medical professionals, ranging from physicians and nurses to medical students, while the target audience of NeuroBot is the general public and patients. Ultimately, NeuroBot has to be easily accessible, readable, and understandable to individuals in the general public by providing responses in layman’s language. It also needs to accommodate users with varying levels of technological proficiency, ensuring usability even for those less familiar with technology. These factors are essential to the success and performance of NeuroBot and should be thoroughly explored in future studies. Future research should involve recruiting real patients from our neurosurgical center to assess their experiences with NeuroBot during trial use. Usability testing and structured feedback from real patients—particularly those from our neurosurgical center—should be prioritized. This includes understanding the experiences of those who may disengage from using the system. A randomized controlled trial could also be conducted to evaluate NeuroBot’s impact on patient outcomes, including its effectiveness in enhancing disease management and improving patient knowledge about their conditions. These user-centered evaluations will be essential to ensure the NeuroBot’s broader acceptability and success in clinical practice.

Before deploying LLM chatbots to public medical settings, ethical compliance must be rigorously examined. Fournier-Tombs and McHardy [32] investigated the ethical aspect of medical conversational AI and listed out a list of potential ethical risks in conversational chatbots, including discrimination, stereotyping, exclusion, lack of privacy, poor data governance, stigma, error tolerance, overconfidence, and technological solutionism. These risks must be systematically investigated and addressed to ensure NeuroBot’s safety and reliability for public use. Moreover, there are potential medicolegal considerations. If patients interpret chatbot-generated suggestions as definitive medical suggestions, it could lead to potential harm. As such, deploying chatbot in clinical settings should establish safeguards mechanisms such as human oversight, legal disclaimers, and protocols for chatbot data monitoring.

One major current concern regarding LLMs is the issue of hallucinations, where a model undertrained in knowledge regarding the prompt will try to guess the correct response and then present the guessed response as a fact in the output, which can colloquially be described as “being confidently wrong” [33]. Such behavior is unacceptable in the medical field, where such a mistake or oversight can irreversibly cause harm to patient safety. Our study attempted to develop a domain-specific model using the RAG approach that helps with mitigating the issue of hallucinations [34]. By supplying relevant factual information along with the prompt to the LLM through RAG, the model will be able to take the factual information into account and generate a more personalized response compared to a nondomain-specific LLM. Our subgroup analysis revealed that prompts that yield the more inaccurate responses are usually in the preoperative and wound-related areas. This result can be attributed to several factors, namely the variability of wound condition from patient circumstances, potential gaps in wound care understanding among providers, and complexity of expectation as wound management could be subjective after several procedures. While we did try to include as many accurate sources as possible of information for every area, our dataset for these 2 areas is comparatively smaller than other areas. This problem of comparative inaccuracy in these 2 areas can be solved by including more sources of knowledge in RAG embedding vector storage for these 2 areas in subsequent studies.

OpenAI researchers Lee et al [35] revealed that the current barebones GPT-4 model is prone to hallucinations from certain prompts and concluded that the question regarding what is considered the acceptable performance of general AI in the medical field remains to be answered. Thus, from both ethical and medicolegal perspectives, disclaimers should always be included in the response or application interface to remind the user that they are interacting with an LLM, and they should seek help from medical practitioners if they have any more concerns or any alarming situations. Some of the phrases our model may reply along with medical information and advice include “It’s important to follow your doctor’s advice as recommendations may vary depending on the specifics of your condition,” “It’s essential to discuss any concerns you have with your doctor to address them and ensure the best possible outcome for your surgery,” “Always consult with your healthcare provider before engaging in any strenuous activities post-stent placement,” or “Do not hesitate to contact your healthcare provider or seek emergency medical care for further assessment and appropriate management.”

A concern of training a highly specific medical chatbot is erroneous or inappropriate output to nonneurosurgical prompts, which is particularly concerning if users inquire about their mental health concerns. Coghlan et al [36] identified that there are quite a few aspects of ethical considerations regarding the use of LLM for mental health assistance purposes. This is because current LLM often cannot grasp the nuances of social, psychological, and biological factors that cause the user’s mental health issues. Thus, misalignment of the AI system with the societal bioethical principles of “autonomy, beneficence, nonmaleficence, justice” may lead to the chatbot to fail at providing a satisfactory response to the user’s query, which may cause harm to the user, which is particularly detrimental for mental health issues. While our chatbot’s domain is primarily limited to neurosurgery, the field of neurosurgery involves diseases regarding the nervous system where patients will often present with highly concerning symptoms and highly affected activities of daily living. Central nervous system surgeries are also ultramajor surgeries, and the prospect of operating on the brain may cause a lot of stress and anxiety for the patients [37-39]. In fact, our qualitative evaluation revealed that while NeuroBot provided responses in a sufficiently positive and professional tone, some participants advised that more empathy is needed in the tone of the response. Thus, while NeuroBot is not required to provide therapist-level mental health advice to the patient, it is paramount for it to be at least safe and be able to respond to the patient appropriately regarding their disease-related anxiety or depression and guide the patient to seek professional help if necessary.

Another feasible direction to improve the safety of NeuroBot is to produce a response validation AI to evaluate and validate the output response to ensure the response is valid and correct before outputting it to the end user. Generative AI is notoriously more difficult to harness than processing and validating a sentence. By creating a domain-specific natural language processing model and integrating it with medical knowledge databases, the pipeline can assign an accuracy score to the output from the generative AI. The system will only deliver the generated response to the patient if the accuracy score meets a predefined threshold, ensuring that the response is sufficiently accurate.

In future studies, we will provide NeuroBot with an easily operable interface, where the system administrator can easily provide the model with up-to-date information. Medicine is a fast-evolving field. During the training of NeuroBot, some protocols were updated, and new research was published. In the qualitative evaluation of the final version of NeuroBot, some participants noted that certain information was outdated. Therefore, it is essential, particularly for long-term maintenance after deployment, to have a system in place that allows medical professionals to update the NeuroBot with the latest information, ensuring that the LLM remains aligned with the evolving medical field.

### Conclusions

This study highlights the potential of NeuroBot as a clinically reliable chatbot to support neurosurgical patients throughout their perioperative journey.

NeuroBot was successfully developed to provide patient-specific, reliable, and comprehensive perioperative education. By leveraging LLMs with RAG technology, NeuroBot demonstrated adaptability in delivering evidence-based responses in perioperative care in neurosurgery. In addition, quantitative evaluations of NeuroBot indicated strong performance in terms of accuracy, relevance, and completeness, with scores consistently above thresholds set during internal and external validations. The Assistants API emerged as the best-performing model, with domain-specific fine-tuning ensuring reliable outputs. Moreover, qualitative analyses of focus group discussions with neurosurgical domain experts revealed high acceptance of NeuroBot as an effective tool for patient education and support. Experts acknowledged its capacity to empower patients, enhance shared decision-making, and streamline routine communication in clinical practice.

NeuroBot represents a significant step forward in integrating AI into patient-centered neurosurgical care. Its validated performance and positive reception by health care professionals underscore its potential to enhance patient education, improve treatment compliance, and reduce health care staff workload.

As a next step, we plan to conduct a prospective randomized controlled trial to evaluate the clinical effectiveness of NeuroBot in a real-world neurosurgical setting. This trial will use a randomized controlled trial to assess reductions in patient anxiety, improvements in disease-related knowledge, and compliance with perioperative instructions. Secondary outcomes will include rates of postoperative complications and hospital readmissions. In addition, qualitative interviews will be conducted to explore patient and provider acceptability, usability, and overall user experience. These findings will guide further refinement and implementation of the chatbot for broader clinical use.

This study provided evidence on NeuroBot as a pioneering example of how LLM-driven applications can improve the quality of care and foster innovation in medical education and patient support. Through continued research and iterative development, NeuroBot can be positioned as a scalable, ethical, and impactful tool in the evolving landscape of health care AI.

## Appendix

Multimedia Appendix 1 Semistructured interview guide.

Multimedia Appendix 2 Thematic analysis from the domain expert.

## Abbreviations

AI: artificial intelligence
LLM: large language model
RAG: retrieval-augmented generation

## Data Availability

All data generated or analyzed during this study are included in this published article (Multimedia Appendices 1 and 2). However, the database of the chatbot is not publicly available, and it will not be provided in this paper.
