# Supplementary material for: Development and Validation of a Large Language Model–Powered Chatbot for Neurosurgery: Mixed Methods Study on Enhancing Perioperative Patient Education
Source: J Med Internet Res. 2025 Jul 15;27:e74299. doi: 10.2196/74299 (PMC12308165; doi:10.2196/74299)
Supplement: Multimedia Appendix 1 [file jmir_v27i1e74299_app1.docx]

**Appendix 1: Semi-Structured Interview Guide**

**Topic 1: General Perceptions of the NeuroBot**

1. **Overall Impressions:**
   - "What are your overall impressions of the NeuroBot?"
2. **Features & Differentiation:**
   - "What features of the NeuroBot do you find most impressive?"
   - "In what ways does the NeuroBot differ from traditional methods you currently use in perioperative education?"
3. **Adoption & Acceptance:**
   - "How open are you, to adopting new technologies like the NeuroBot in your practice?"
   - "What factors do you think contribute to the acceptance of such technology in your setting?"

**Topic 2: Usability and Integration into Routine Care**

1. **Ease of Use:**
   - "How would you rate the ease of learning and using the NeuroBot? Could you share specific experiences?"
   - "What aspects of the NeuroBot’s interface or design support or hinder its usability?"
2. **Operational Challenges:**
   - "What challenges would you expect to encounter when using the NeuroBot during your shifts?"
   - "Are there any features or functions that you feel could be improved to enhance its integration into daily care?"

**Topic 3: Accuracy and Relevance in Clinical Practice**

1. **Clinical Accuracy:**
   - "How accurate do you find the NeuroBot’s outputs in your clinical practice?"
2. **Relevance to Patient Management:**
   - "In your experience, how relevant is the information provided by the NeuroBot to your day-to-day patient management?"
   - "Are there any areas where you think the NeuroBot’s clinical relevance could be improved or expanded?"
3. **Reliability & Trust:**
   - "How confident are you in using the NeuroBot for perioperative patient care support?"
   - "Have you observed any discrepancies between the NeuroBot’s suggestions and actual situatiom?"

**Topic 4: Potential Impact on Patient Care**

1. **Patient Outcomes:**
   - "In what ways do you believe the NeuroBot has the potential to improve patient outcomes?"
2. **Quality of Care:**
   - "How might the NeuroBot enhance the quality or safety of care provided to patients?"
   - "Are there particular patient groups or conditions that you think would benefit most from using the NeuroBot?"
3. **Efficiency and Timeliness:**
   - "Do you feel that the NeuroBot can help in reducing delays or improving the efficiency of care delivery? How?"
   - "What impact do you see the NeuroBot having on your team’s ability to respond to patient needs promptly?"

**Topic 5: Challenges and Recommendations**

1. **Identified Challenges:**
   - "What challenges have you experienced or observed with the NeuroBot in your practice?"
   - "Are there any technical or operational barriers that limit its use?"
2. **Improvement Suggestions:**
   - "What recommendations would you make to improve the usability and functionality of the NeuroBot?"
   - "Are there any additional features you believe would make the NeuroBot more valuable in your clinical practice?"
3. **Future Integration:**
   - "How do you see the role of the NeuroBot evolving in your clinical setting over the next few years?"
   - "What strategies could be employed to encourage broader adoption and smoother integration of the NeuroBot into routine care?"
