# Supplementary material for: Development and Validation of a Large Language Model–Powered Chatbot for Neurosurgery: Mixed Methods Study on Enhancing Perioperative Patient Education
Source: J Med Internet Res. 2025 Jul 15;27:e74299. doi: 10.2196/74299 (PMC12308165; doi:10.2196/74299)
Supplement: Multimedia Appendix 2 [file jmir_v27i1e74299_app2.docx]

**Appendix 2: Thematic Analysis from Domain Expert**

1. **NeuroBot use as a patient resource tool**

All participants expressed potential use for a chatbot would be to use it as a patient resource tool that a neurosurgical patient could interact with.

*“It’s helping them by providing prompt, validated and reliable information with 24/7 availability. We and doctors are always busy…for some general questions, like visiting hours, when to resume diet after surgery…I think NeuroBot can help…”*

(Doctor, participant h, in practice for 11 years)

Some participants observed that patients often spend time on searching potentially inaccurate internet sources, and believe chatbots can quickly provide reliable information, enhancing patient access to accurate knowledge.

*"Patients often turn to the internet when they need more information about their illness, but the information may not be reliable, and it also takes time and effort for them to search … A chatbot can help by delivering accurate information almost instantly, saving patients the time and effort of searching on their own."*

(Doctor, participant 1, in practice for 4 years)

There are few participants who have verbalised that NeuroBot facilitates patients by generating personalised patient education content, tailored to varying levels of health literacy.

*“It is invaluable for creating tailored education that meets patients at their level of understanding and language…I tried to ask for clarification in some terminologies, and I found that it instantly regenerated responses using more simplified words and with more explanation”*

(Doctor, participant c, in practice for 9 years)

1. **NeuroBot use as a healthcare tool for enhancing patient health outcomes**

Participants believed that by delivering crucial, condition-specific surgical information accessible to families and caregivers, NeuroBot helps improve their understanding of the illness and alleviates stress.

*"Patients and their families often feel really confused and worried about what will happen during and after surgery. NeuroBot can give them the information they need, which helps calm their nerves and makes the whole process a lot clearer for them."*

(Nurse, participant a, in practice for 8 years)

Participants noted that NeuroBot provided crucial information on lifestyle modification advice, helping patients and their caregivers dispel myths and misconceptions, ultimately improving surgical outcomes.

"*I once had a patient who, after carotid stent placement, took part in strenuous exercise, like jogging or swimming, and the puncture site got a big lump after that…He said he misunderstood he can return to normal life after discharge. This highlights the importance of using digital tools that can provide regular notifications at different stages, providing self-care tips and reinforce important information as reminders from time-to-time."*

(Nurse, participant J, in practice for 12 years)

Participants observed that patients often feel overwhelmed by the challenges of surgery, perceiving themselves as inadequately prepared for the procedure and potential postoperative complications. An easily accessible NeuroBot could support patients throughout their surgical journey, enhancing their self-efficacy and better equipping them for the surgery.

*"Patients often feel helpless before surgery, not knowing much about how to prepare or handle things that might come up after they’re discharged. NeuroBot could really help by offering preoperative tips and advice for when they’re back home, empowering them to manage their health."*

(Nurse, participant a, in practice for 8 years)

1. **Chatbot use as a patient- healthcare professional communication tool**

Participants observed that the anonymity provided by NeuroBot creates a psychologically safe environment for patients or caregivers  to disclose personal concerns, increasing their willingness to seek help when needed.

*"I once had a patient with glioblastoma. The patient wanted to know more about hospice and palliative care arrangements, but he said he was not dare to ask such issues in front of parents and his wife during visiting hours, and wanted to ask me personally..."*

(Nurse, participant p, in practice for 4 years)

Interestingly, four junior nurses, who have experience less than 6 years, found NeuroBot helpful for simplifying neurosurgical terms, making it easier for them to answer patient and family questions effectively.

*"I often struggle to explain medical terms like arteriovenous malformation or cavernoma to patients and their families. We learn these terms in English, and it’s tough to translate them into Cantonese in a way that’s easy to understand. Patients and families often end up confused after my explanations. I’m really impressed with NeuroBot—it helps me explain these terms much more precisely in a minute."*

(Nurse, participant k, in practice for 1 year)

Most nurses (10 out of 11) believe that NeuroBot is useful for handling general patient inquiries, such as hospital visiting hours and general perioperative questions, freeing up healthcare staff to focus on more complex patient needs.

*"I often have to deal with a flood of questions from patients and caregivers over the phone or during visiting hours. I think NeuroBot could save me time by handling general patient inquiries, allowing me to focus more on bedside nursing care."*

(Nurse, participant m, in practice for 3 years)

Some participants observed that patients and families often struggle to communicate effectively with physicians or feel uncomfortable speaking with doctors face-to-face. They believe that NeuroBot could bridge this gap by acting as a round-the-clock, healthcare staff-like assistant, addressing some of their questions and making them feel more cared for.

*"My patients often tell me they don't even know who their doctor is, or they can’t think of questions to ask during their appointment. But as soon as the doctor leaves, they remember what they wanted to ask, and it's too late. I think the bot could help bridge that communication gap…”*

(Nurse, participant q, in practice for 24 years)

1. **Perceived positive features of NeuroBot**

Most participants (17/18) preferred NeuroBot's simplicity and user-friendly design, mimicking common the design of commonly used messaging apps, which benefits those with limited tech-savviness.

*"It's really easy to use, which is great for people who aren’t familiar with chatbot… It just looks like using “We-chat”, most people can definitely use it without any trouble."*

(Doctor, participant d, in practice for 21 years)

Two- third of participants appreciated NeuroBot’s natural language comprehension feature, which allows users to ask questions in their own words and receive relevant, tailored information without needing to use specific predefined keywords like rule-based chatbots.

*"I really like that NeuroBot uses natural language processing. Unlike those customer-service chatbots that require you to use specific keywords, the bot often understands what I'm asking and gives me the information I need."*

(Doctor, participant r, in practice for 10 year)

All participants found that NeuroBot delivers information quickly and is patient- centered, making it well-suited as a patient support tool.

*"The answers are clear and easy to understand from a patient’s perspective. It provides enough information to cover the usual questions patients frequently ask, which is perfect as an information tool."*

(Nurse, participant p, in practice for 4 years)

1. **Perceived cons or concerns of NeuroBot**

Some participants (3/18) think that the design of NeuroBot is elementary and the colour and layout are too simple and not attractive.

*"It would be more engaging if users could choose background colours, like those instant messaging apps...Now, it looks a bit too plain and serious."*

(Nurse, participant f, in practice for 11 years)

Some participants (4 out of 18) suggested that the response layout would benefit from point form and bolded key information, making it easier for users to quickly grasp the main points.

*"The info comes in long paragraphs, which can be hard to read. Having key points in bold would make it much easier to get the important details quickly."*

(Nurse, participant a, in practice for 8 years)

More than half of the participants (10 out of 18) felt that the dialog box for input is too small, and that text-based input might be challenging for some users and suggesting adding the feature of multimodal conversations.

*"The dialogue box for text input is quite small and takes up only a small part of the interface. It can be tough for some users to type on a mobile phone keypad, as well as reading the text that showed on such tiny screen. Adding features like voice input and output with voice and video, in addition to text would be better."*

(Nurse, participant f, in practice for 11 years)

More than half of participants (12 out of 18) concerned that the general public might still struggle to accept and effectively use the chatbot.

*“I think many patients and families might have concerns... They often prefer interacting with real people and might find it difficult to accept talking to a robot."*

(Nurse, participant e, in practice for 10 years)

*“ You may consider incoporating the NeuroBot into those commonly used digital platforms like We-Chat or WhatsApp, which are readily available and familiar to most users, especially elderly.”*

(Doctor, participant g, in practice for 6 years)

Nearly one- third of participants (5 out of 18) raised doubts about the possibility that inaccurate or outdated information will be provided. They worried this could lead to negative patient outcomes and liability issues. Some participants suggested adding disclaimer to protect both users and developers, maintaining ethical and legal boundaries.

*"I still don't understand how it generates answers. Providing inaccurate answers on life-and death issues, such as misdiagnosing a complication, could have disastrous consequences for patients. And if the information is wrong, who will be responsible?"*

(Doctor, participant d, in practice for 21 years)

Nearly half of the participants (7 out of 18) expressed concerns that the rapid pace of changes in medical knowledge and technology means NeuroBot would require frequent updates to ensure the information it provides remains current.

*"Medical knowledge evolves fast—what's new today could be outdated in just a few years. It's a big challenge to make sure the bot provides up-to-date information consistently."*

(Doctor, participant g, in practice for 6 years)

Considering the future of AI in healthcare, all participants agreed on recommending NeuroBot to their patients as a supplementary resource tool alongside routine neurosurgical care.

*"We're in a digital health era where everything needs to be timely and accurate. I'm not worried about AI tools replacing us. Instead, I see it more like a co-pilot... a trusted companion for both patients and us."*

(Nurse, participant b, in practice for 10 years)
